# Threat-Avoidance Tendencies Moderate the Link Between Serotonin Transporter Genetic Variation and Reactive Aggression

**DOI:** 10.3389/fnbeh.2020.562098

**Published:** 2020-09-28

**Authors:** Deborah G. A. Peeters, Wolf-Gero Lange, A. Katinka L. von Borries, Barbara Franke, Inge Volman, Judith R. Homberg, Robbert-Jan Verkes, Karin Roelofs

**Affiliations:** ^1^Department of Cognitive Neuroscience, Donders Institute for Brain, Cognition and Behaviour, Radboud University Medical Center, Nijmegen, Netherlands; ^2^Department of Psychiatry, Donders Institute for Brain, Cognition and Behaviour, Radboud University Medical Center, Nijmegen, Netherlands; ^3^Department of Clinical Psychology, Radboud University Nijmegen, Nijmegen, Netherlands; ^4^Behavioural Science Institute, Radboud University Nijmegen, Nijmegen, Netherlands; ^5^Department of Psychocardiology, Clinic for Internal Medicine and Cardiology, Bergmannsheil und Kinderklinik Buer GmbH, Gelsenkirchen, Germany; ^6^Institute for Stressmedicine, ISM Rhein Ruhr, Gelsenkirchen, Germany; ^7^Department of Human Genetics, Donders Institute for Brain, Cognition and Behaviour, Radboud University Medical Center, Nijmegen, Netherlands; ^8^Department of Psychiatry, University of Oxford, Oxford, United Kingdom; ^9^Wellcome Centre for Integrative Neuroimaging, Centre for Functional MRI of the Brain, Nuffield Department of Clinical Neurosciences, John Radcliffe Hospital, University of Oxford, Oxford, United Kingdom; ^10^Donders Institute for Brain, Cognition and Behaviour, Radboud University Nijmegen, Nijmegen, Netherlands

**Keywords:** serotonin, 5-HTTLPR, aggression, approach-avoidance task, Taylor aggression paradigm

## Abstract

The short (S) allele of the serotonin transporter-linked promoter region (5-HTTLPR) polymorphism has been linked to reactive aggression in men, but this association is less consistent in females. Reactive aggression has been particularly described as a result of fear-driven defense to threat, but how this interaction between defensive behavior and aggression is expressed in S-allele carriers remains unknown. In order to explore this interplay between 5-HTTLPR genotype, defensive behavior and reactive aggression, we combined genotyping with objective measures of action tendencies toward angry faces in an approach-avoidance task (AAT) and reactive aggression in the Taylor aggression paradigm (TAP) in healthy females, *N* = 95. This study shows that female S-allele carriers in general display increased implicit reactive aggression (administering aversive white noise) toward opponents. Furthermore, we found that threat-avoidance tendencies moderate the association between 5-HTTLPR genotype and aggression displayed on the TAP. Together, these findings indicate a positive correlation between avoidance of angry faces in the AAT and reactive aggression in the TAP exclusively present in S-allele carriers.

## Introduction

Although aggressive behavior is an evolutionary conserved social behavior, uncontrolled aggression has a huge adverse impact on society. Among several candidate mechanisms, the serotonergic system seems to play an important role in developing aggressive behavior. A potential mediating factor in this is a functional genetic polymorphism within the promotor sequence of the serotonin transporter gene (5-HTT, official name SLC6A4). The short (S) allele is associated with reduced transcription of the 5-HTT mRNA compared to the long (L) allele variant, resulting in less transporters and consequently decreased serotonin reuptake, increased synaptic serotonin levels and an adaptive decrease in serotonergic neurotransmission (Gregory et al., 1998; Hanna et al., 1998; Greenberg et al., 1999; Lesch, 2001). Low cerebrospinal fluid (CSF) levels of the serotonergic metabolite 5-HIAA are associated with aggression, and selective serotonin reuptake inhibitors have shown anti-aggressive effects (Brown et al., 1979; Linnoila et al., 1983 for reviews see, Duke et al., 2013; Coccaro et al., 2014). More specifically, reactive aggression, as opposed to instrumental aggression, has been associated with low serotonin levels (Glenn, 2011; Coccaro, 2012; Montoya et al., 2012) and genetic factors linked to serotonin function and metabolism (e.g., Waltes et al., 2016; van Donkelaar et al., 2017).

Aggression often is part of a multifaceted behavioral profile, making it difficult to show direct genotypic associations. Nevertheless, an indirect association with the serotonin transporter genotype has been observed in behavior associated with reactive aggression. Externalizing behaviors that are often linked to reactive aggressive behavior such as antisocial personality disorder, conduct disorder and attention-deficit/hyperactivity disorder (ADHD) seem to be all associated with increased frequency of the S-allele in mainly male populations (Cadoret et al., 2003; Sakai et al., 2006; Retz and Rösler, 2009). However, meta-analyses attenuate these findings for ADHD (Gizer et al., 2009; Landaas et al., 2010; Lee and Song, 2018). Furthermore, the S-allele is more prevalent in violent alcohol and heroin dependent males as opposed to non-violent males (Hallikainen et al., 1999; Gerra et al., 2005) as well as in violent forensic psychiatric male patients (Retz and Rösler, 2009). However, these relations are less clear in females, where the literature seems to be even more contradictory. In contrast to men, females carrying the S-allele showed lower levels of symptom counts for conduct disorder, aggression, and ADHD behavioral scales (Cadoret et al., 2003). However, they showed higher levels of aggression and hostility in the Buss-Durkee Hostility Inventory and anxiety on the State-Trait Anxiety inventory (Gonda et al., 2009). The latter observation is in line with notions that reactive aggression may be a manifestation of fear-driven defense-systems (Blair, 2016).

Indeed, whereas instrumental aggression, such as observed in psychopathy, is linked to fearlessness (Blair, 2016), more reactive forms of aggression have been linked to increased anxiety, arousal, and negative affect (Hallikainen et al., 1999; Cadoret et al., 2003; Gerra et al., 2005; Sakai et al., 2006; Gonda et al., 2009; Retz and Rösler, 2009). Although it is assumed that fearful threat-avoidance reactions underlie overt reactive aggression, this link has never been demonstrated directly. Indirect evidence stems from studies investigating approach-avoidance action tendencies using the approach-avoidance task (AAT). A series of studies using an AAT showed that, whereas reduced fearful avoidance tendencies to angry faces are found in psychopathy (von Borries et al., 2012), increased avoidance is linked to anxiety (Heuer et al., 2007; Roelofs et al., 2010). Second, recent neuroimaging work indicated that S-allele carriers seem to have reduced prefrontal control over the amygdala, when they have to control and reverse their approach-avoidance action tendencies on the AAT (e.g., switch from approach happy to avoid happy) (Volman et al., 2013). However, it remains to be tested, whether changed approach-avoidance tendencies in S-allele carriers indeed moderate increased reactive aggression.

In the present study, we set out to test, whether increased reactive aggression in healthy female S-allele carriers is moderated by approach-avoidance tendencies. We expected that S-allele carriers compared to L-allele homozygotes would show increased reactive aggression on the Taylor aggression paradigm (TAP), a well-established laboratory aggression provocation task in which participants select the intensity and duration of white noise that they can deliver to a provoking opponent. In addition, we expected that this relation would be moderated by the increased tendency to approach angry faces in the AAT.

## Materials and Methods

### Procedure

After indicating interest, participants were sent a link to an online-survey platform^1^. There, they were informed about the study and asked to sign an online informed consent form. Then, participants filled in a number of questionnaires at home, under which sociodemographics (e.g., age, proficiency in Dutch language, etc.), the RPQ and IPAS (see section “Questionnaires”) and other questionnaires not relevant for the current study [i.e., SRP (self-report psychopathy scale; Williams et al., 2003), TriPM (triarchic psychopathy measure; Patrick, 2010), PPI (psychopathic personality inventory; Lilienfeld and Andrews, 1996), and LSAS (Liebowitz social anxiety scale; Liebowitz, 1999)]. After the completion of the first set of questionnaires at home, they were invited to the lab where they signed another informed consent form for the rest of the study and completed another set of questionnaires online non-relevant for the current study [i.e., STAXI (state trait anger expression inventory; Spielberger, 1996), STAI (state trait anxiety inventory; Spielberger, 1983)]. A summary of these scores can be found in Supplementary Table 1. Subsequently, a set of saliva samples was collected to enable genotyping (see section “Genotyping”). Then, participants completed the AAT (see section “Approach-Avoidance Task (AAT)”) followed by the TAP (2,5,1). Finally, participants were debriefed, compensated, thanked for their effort and dismissed.

### Participants

From a sample of 239 female students taking part in a bigger research project, 95 female students were included in the present study at Radboud University in Nijmegen, Netherlands. They agreed to provide saliva sampling for genotyping and completed the TAP (*N* = 95). Their mean age was 20.09 (± SEM 0.259) and their main educational program was Psychology (Psychology, *N* = 55; Science of teaching, *N* = 11, Miscellaneous, *N* = 29). Ten participants showed missing data in the at home questionnaires (*N* = 85) and three participants showed missing data in the AAT (*N* = 92). These could not be taken into account for the corresponding analyses. This study was approved by the local ethical review board (CMO region Arnhem-Nijmegen; NL42229.091.12). Participants gave written informed consent and received either course credits or €20 for maximal 3 h of participation.

### Genotyping

Genotyping of the serotonin transporter-linked promoter region (5-HTTLPR) was performed using polymerase chain reaction according to standard protocols at the Department of Human Genetics of the Radboud University Medical Center in Nijmegen, Netherlands. After the PCR, fragment length analysis was performed on the ABI Prism 3730 Genetic Analyser (Applied Biosystems, Nieuwerkerk aan den IJssel, Netherlands), and results were analyzed with GeneMapper Software, version 4.0 (Applied Biosystems).

### Questionnaires

To enable description of participants’ aggressive behavior in terms of commonly used, standardized measures, two self-report questionnaires were used: the reactive proactive aggression questionnaire (RPQ; Raine et al., 2006) and the impulsive premeditated aggression scale (IPAS; Stanford et al., 2003). Both questionnaires evaluate proactive and reactive aggression during the last 6 months. In the RPQ the proactive subscale consists of 12 items (e.g., “How often have you had fights with others to show who was on top?”), and the reactive subscale consists of 11 items (e.g., “How often have you reacted angrily when provoked by others?”). Items on the RPQ are rated on a three-point scale ranging from 0 (never) to 2 (often). The original RPQ as well as the Dutch version have proven sufficient psychometric validity and reliability (Cima et al., 2013; Brugman et al., 2017). In the IPAS, the impulsive subscale consists of 10 items (e.g., “When angry, I reacted without thinking”), and the premeditated subscale consists of eight items (e.g., “I think the other person deserved what happened to them during some of the incidents”). Items on the IPAS are rated on a five-point Likert scale ranging from 0 (strongly disagree) to 5 (strongly agree). The original IPAS as well as the Dutch version have proven psychometric validity and reliability (Kuyck et al., 2013). Both the RPQ and IPAS were completed by eighty-five participants.

### Tasks

#### Taylor Aggression Paradigm (TAP)

A modified version of the TAP (Taylor, 1967) was used to measure aggression. Participants were told that they were competing in a reaction time task against a male opponent who they met in the waiting room before being shown into adjacent rooms, while in fact there was no opponent. All participants were asked whether they noticed anything out of the ordinary, and none of them reported that they did not believe the set up.

They were told that they could determine before each trial the severity of the punishment that the opponent should receive when she would lose, by setting the intensity [ranging from 1 (5 dB) to 10 (100 dB)] and the duration [ranging from 1 (500 ms) to 10 (5000 ms)] of a burst of white noise that the loser would hear via headphones. Once the task was started and the punishment values for the next trial were set, a square appeared on the screen. As soon as the square turned green, the participant was supposed to click on that square as fast as possible although winning and losing trials were programmed beforehand and is held constant for all participants (i.e., each participant wins and loses half of the trials). In total they completed 30 trials divided into 3 blocks of 10. Stimuli presentation and acquisition of responses were controlled by a PC running Presentation software. In the first seven trials, the participant would win and her presets for the punishment of the “loser” were seen as indices of instrumental aggression. From the 8^*th*^ trial on the task was programmed to let the participant lose on a chance level. Participants’ choices for punishment values after having been punished themselves, were seen as indices of reactive aggression. The experiment had a duration of 9 min.

#### Approach-Avoidance Task (AAT)

Stimuli were black and white pictures (sized 8.4 cm × 13.5 cm) of facial expressions (eight actors: four female, four male) showing different facial expressions (angry, happy, neutral) selected from Ekman and Friesen (1976) and Karolinska Institute face databases (Lundqvist et al., 1998). Eye regions were modified to show either a direct or an averted gaze (left or right). This resulted in 48 different stimuli (8 actors ^∗^ 3 facial expressions ^∗^ 2 gaze directions) (Roelofs et al., 2010). Here, only direct gaze pictures were analyzed as these are relevant in provocation of aggressive behavior and therefore relevant to associate with the TAP.

Pictures were presented on a computer screen (resolution 1024 × 768 pixels). Participants were seated about 60 cm from the screen with a joystick (Logitech Attack 300) in front of them. The joystick could be pushed or pulled by moving their arm toward (approach) or away (avoid) from their body. This joystick movement resulted in the picture growing (approach) or shrinking (avoid) in size (size in degree of visual angle: start: 9.5°^∗^13°; minimal: 3.5°^∗^4.5°; maximal: 15.5°^∗^20°). This zooming version (Rinck and Becker, 2007) is resistant to possible cognitive re-interpretation of arm-movements [i.e., pushing/pulling unambiguously mean avoidance/approach, respectively (von Borries et al., 2012)]. Stimuli presentation and acquisition of joystick positions were controlled by a PC running Presentation software.

In each of six pre-randomized blocks (instructions: pull neutral – push angry, push neutral – pull angry, pull neutral – push happy, push neutral – pull happy, pull happy – push angry, push happy – pull angry) 64 experimental trials (32 straight gaze, 32 averted gaze) were presented. Each block was preceded by 16 practice trials in which a trial could only be ended when the correct movement was executed, while no feedback was provided in the experimental trials. All trials were presented in a pre-randomized order with no more than three of the same stimulus response combinations presented successively. Six different block orders were assigned to participants in a pre-randomized way. After each block, participants were given the option to take a short, self-paced break.

Each trial was completed when the joystick was moved the maximum of 30° (in pull or push direction) and the picture disappeared from the screen. Subsequently, the participants had to return the joystick to a neutral (upward) position and had to press the ‘fire button’ to proceed to the next trial, making each trial self-paced.

Participants were instructed to respond as fast and accurate as possible. Reaction time (RT) from appearance of the stimulus to the correct maximum movement of 30° was used to measure approach and avoidance tendencies. The experiment had a duration of 20 min.

### Statistical Analyses

In the TAP, to combine measures of selected white noise intensity and duration in single aggression scores the average selected white noise intensity and duration were transformed into standardized z-scores and summed per phase in the TAP (i.e., average across all trials (TAP total); average across trials 1–7 before provocation (TAP before provocation), average across trials 8–25 after provocation (TAP after provocation) and trial eight immediately after provocation (TAP provocation) (Dambacher et al., 2015a,b). Spearman correlations for the subscales of reactive and instrumental aggression in the RPQ, IPAS and TAP can be found in Supplementary Table 2.

The Hardy-Weinberg equilibrium of the 5-HTTLPR genotype was examined using the Chi-square test for goodness of fit (Emigh, 1980). All data were checked for outliers and normality (using the Shapiro–Wilk test). One-way analyses of variance (ANOVAs) were used to compare between genotypes (S-allele carrier vs. L-allele homozygote). When the data were non-normally distributed, non-parametric Mann–Whitney *U*-tests were used to compare between groups. This strategy of comparing between genotypes was used in the descriptives, TAP and AAT.

According to a wealth of previous work on the 5-HTTLPR genotype (e.g., Bondy et al., 2000; Cadoret et al., 2003; Pezawas et al., 2005; Marsh et al., 2006; Heinz et al., 2007; Gonda et al., 2009), including our own on the relation between 5-HTTLPR polymorphisms and AAT (Volman et al., 2013), we investigated the effect of 5-HTTLPR genotype on aggression on the TAP total variable. Next, following previous work with the TAP (Elson et al., 2014), we explored TAP aggression in the other phases (TAP before provocation, TAP after provocation and TAP provocation). Further testing explored whether observed group differences were driven by the white noise intensity, duration or both.

In the AAT, mean RTs for correct responses per facial expression (angry, happy, neutral) and movement (approach, avoid) were calculated. Correct responses were filtered using a < 150 and > 1500 ms reaction time (RT) cut-off (von Borries et al., 2012). Following previous work, these values were used to calculate AAT effect scores by subtracting mean pull RTs from corresponding mean push RTs (i.e., AAT anger: angry push – angry pull; AAT happy: happy push – happy pull; AAT neutral: neutral push – neutral pull). In this way AAT effect scores represent a relative avoidance/approach tendency (Roelofs et al., 2010). One sample t-tests were used to see if the effect scores differed from 0.

Next we examined whether avoidance tendency toward angry faces would moderate the interaction between 5-HTTLPR genotype and aggression provoked by the TAP provocation. For this, we used multiple regression analyses. First the individual continuous AAT effect scores for angry faces were centered by converting them into z-scores, as recommended by Aiken et al. (1991). Next, the interaction term was calculated by obtaining the cross-product between the dummy-coded 5-HTTLPR genotype and standardized AAT effect score for angry faces (AAT anger). Parametric Pearson correlations (for normally distributed variables) were used to investigate the correlation between aggression in the TAP provocation and AAT anger per genotype (S-allele carrier and L-allele homozygote) and Fisher’s transformations of those correlation coefficients were used to assess the significance of difference between them.

All statistical analyses were performed using SPSS version 23 (SPSS, Inc., Chicago, IL, United States). All statistical tests were two-sided and level of significance was set at *p* < 0.05.

## Results

### Descriptives and Serotonin Transporter Gene (5-HTTLPR)

Ninety-five participants were included in the study. The distribution of the serotonin transporter gene polymorphism (5-HTTLPR) followed the Hardy-Weinberg equilibrium, *X*^2^(2, *N* = 95) = 0.003, *p* = 0.999 with: S-allele homozygote (18.9%); S/L allele heterozygote (49.5%) and L-allele homozygote (31.6%). Assuming dominance of effects of the S-allele, consistent with earlier work (e.g., Bondy et al., 2000; Cadoret et al., 2003; Pezawas et al., 2005; Marsh et al., 2006; Heinz et al., 2007; Gonda et al., 2009) as well as our previous work on the AAT (Volman et al., 2013), we compared S-allele carriers with L-allele homozygotes. Descriptives regarding these groups with respect to age and questionnaires are displayed in Table 1. Internal consistency for the RPQ subscales (Cronbach’s alpha = 0.787) and IPAS subscales (Cronbach’s alpha = 0.835) were good.

**TABLE 1:** Mean (standard deviation; SD) age (years) and aggression scores in the reactive proactive aggression questionnaire (RPQ) and the impulsive premeditated aggression scale (IPAS) for participants in the S-allele carrier group and the L-allele homozygote group of the serotonin transporter-linked promoter region (5-HTTLPR) genotype.

	5-HTTLPR genotype	
	
	S-allele carriers (*N* = 55)	L-allele homozygote (*N* = 30)
Age (years)	20.20 (2.57)	19.90 (2.02)
**RPQ**		
Total aggression	6.95 (4.08)	6.07 (3.71)
Reactive aggression	5.98 (3.03)	5.73 (3.34)
Proactive aggression	0.96 (1.39)	0.33 (0.80)
**IPAS**		
Total aggression	43.93 (9.82)	43.00 (7.25)
Impulsive aggression	26.15 (6.35)	24.77 (5.82)
Premeditated aggression	17.78 (5.83)	18.23 (5.69)

### Taylor Aggression Paradigm (TAP)

**FIGURE 1:**
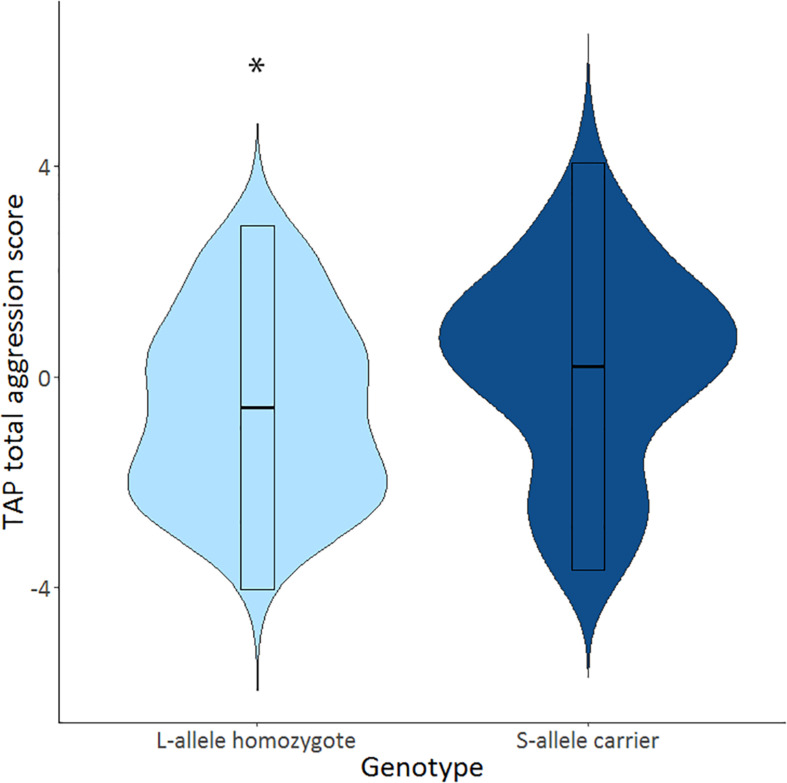
Violin plots show distributions of Taylor aggression paradigm (TAP) total aggression score across all trials (TAP total) for of the serotonin transporter-linked promoter region (5-HTTLPR) L-allele homozygotes (*N* = 30; light blue) and S-allele carriers (*N* = 65; dark blue). Significant genotype effects indicated by ^∗^*p* < 0.05.

The mean selected duration score in the TAP was 2.81 (*SD* = 0.16), and the mean selected intensity score in the TAP was 3.15 (*SD* = 0.18). S-allele carriers showed a higher TAP total score compared to the L-allele homozygotes, *U* = 706.5, *p* = 0.032 (see Table 2 and Figure 1). Explorative tests for the separate phases showed that this effect was significant after the first provocation (TAP after provocation: *U* = 725.0, *p* = 0.045), and trend significant before the first provocation (TAP before provocation: *U* = 743.5, *p* = 0.064) and not in the provocation stage (TAP provocation: *U* = 788.5, *p* = 0.134; see Table 2). Further testing explored the relative contribution of the duration and intensity of white noise in the TAP total score. S-allele carriers as compared to the L-allele homozygotes showed significantly increased selection of duration of white noise averaged across all trials *U* = 679.0, *p* = 0.018. Groups did not differ with respect to the intensity across all trials *U* = 780.0, *p* = 0.118. The increased duration of white noise administered to the opponent was present in all stages (TAP before provocation: *U* = 714.0, *p* = 0.035, TAP provocation: *U* = 732.0, *p* = 0.047 and TAP after provocation: *U* = 698.0, *p* = 0.026). Together, these findings suggest that S-allele carriers show higher aggression scores in the TAP compared to L-allele homozygotes, mainly driven by the increased duration of the white noise delivered to their opponents throughout all TAP stages.

**TABLE 2:** Taylor aggression paradigm parameter means (SDs) for participants in the S-allele carrier group and the L-allele homozygote group of the serotonin transporter-linked promoter region (5-HTTLPR) genotype.

	5-HTTLPR genotype					
	
	S-allele carriers (*N* = 65)			L-allele homozygote (*N* = 30)		
		
	Aggression score	Duration white noise	Intensity white noise	Aggression score	Duration white noise	Intensity white noise
Total TAP	0.27 (1.92)*	3.05 (1.61)*	3.35 (1.76)	−0.59 (1.73)	2.28 (1.37)	2.71 (1.69)
TAP before provocation	0.25 (1.96)	2.13 (1.54)*	2.71 (2.00)	−0.54 (1.41)	1.49 (0.97)	2.08 (1.56)
TAP after provocation	0.26 (1.91)*	3.33 (1.76)*	3.50 (1.82)	−0.56 (1.78)	2.52 (1.57)	2.86 (1.78)
TAP provocation	0.21 (1.90)	2.97 (2.18)*	3.43 (2.13)	−0.45 (1.64)	2.00 (1.46)	3.03 (2.43)

Parameters were split up in separate durations and intensities of white noise selected by the participants. The aggression score is the sum of duration and intensity after transformation into standardized z-scores. Significant genotype effects indicated by *p < 0.05.

### Approach-Avoidance Task (AAT)

The AAT effect scores (mean RTs for push minus pull) for each of the three facial expressions are presented in Table 3. Negative effect scores were found for angry *t*(91) = −4.38, *p* ≤ 0.001 and neutral *t*(92) = −3.59, *p* = 0.001 facial expressions, and both were significantly different from zero. This indicates that participants were faster in avoiding these expressions compared to approaching them. For happy facial expressions the opposite effect was found *t*(92) = 2.09, *p* = 0.040. Considering 5-HTTLPR genotype effects in the AAT, no differences between S-allele carriers and L-allele homozygotes were found on AAT anger *F*(1,90) = 0.08, *p* = 0.774), AAT happy *F*(1,91) = 0.45, *p* = 0.504 and AAT neutral *F*(1,91) = 0.06, *p* = 0.804.

**TABLE 3:** Approach-avoidance task (AAT) reaction time means in ms (SDs) for avoidance (push) and approach (pull) responses to angry, happy, and neutral facial expressions and their corresponding effect scores (= push – pull).

Valence	Push (avoid)	Pull (approach)	Effect score
Angry	724 (10)	749 (11)	−25
Happy	706 (10)	690 (11)	17
Neutral	722 (10)	747 (11)	−25

### The Interplay of 5-HTTLPR Genotype, Aggression, and Avoidance

**FIGURE 2:**
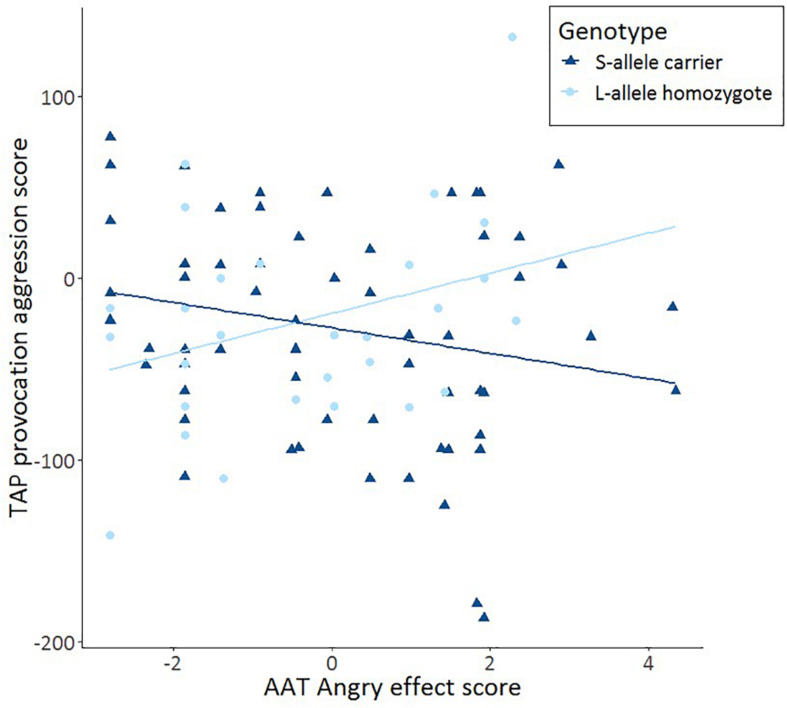
Scatter plots and linear trend lines show the correlations for the approach-avoidance task (AAT) effect score (= push – pull) as a function of TAP aggression score from trial 8 immediately after provocation (TAP provocation) for S-allele carriers and L-allele homozygotes of the serotonin transporter-linked promoter region (5-HTTLPR) genotype.

After having established that S-allele carriers show more aggression on the TAP, we tested our hypothesis that such an effect is moderated by approach-avoidance tendencies. Specifically, we tested whether AAT anger moderates the interaction between 5-HTTLPR genotype and TAP provocation score using a multiple regression analysis. In the first model, 5-HTTLPR genotype and AAT anger were included. These variables together did not account for a significant amount of variance in aggression, *F*(2,89) = 1.195, *p* = 0.307; *R*^2^ = 0.004. In the second model, the interaction term between 5-HTTLPR genotype and AAT anger was added, which accounted for a trend significant amount of variance in aggression, *F*(3,88) = 2.673, *p* = 0.052; *R*^2^ = 0.052. This interaction term accounts for an extra 5.7% of the variance in aggression (*R*^2^ change = 0.057, *p* = 0.021). Using this model, a significant moderation between AAT anger and 5-HTTLPR genotype on the TAP provocation score was found *b* = −0.950, *p* = 0.021. Subsequent tests showed a trend significant effect of S-allele carriers’ threat-avoidance tendencies moderating increased aggression elicited by the TAP *r*(62) = −0.219, *p* = 0.088 and a trend significant effect of L-allele homozygotes’ threat-avoidance tendencies moderating decreased aggression elicited by the TAP *r*(30) = 0.324, *p* = 0.081. Furthermore, the difference between these correlations was statistically significant, *Z* = −2.4, *p* = 0.016 (Figure 2). There were also no moderation effects for happy or neutral faces in S-allele carriers on the TAP (all *p* > 0.18). The data show that AAT anger moderates the link between 5-HTTLPR genotype and TAP provocation. These findings suggest that increased avoidance of angry faces on the AAT is associated with increased reactive aggression on the TAP in 5-HTTLPR S-allele carriers and decreased reactive aggression on the TAP in L-allele homozygotes.

## Discussion

The preliminary findings in this study suggest that the relation between 5-HTTLPR S-allele carrier genotype and reactive aggression is moderated by approach-avoidance tendencies. Using an aggression provocation paradigm in ninety-five female participants we found that S-allele carriers showed increased reactive aggression, expressed through administering aversive white noise to an opponent for a longer duration. Moreover, avoidance tendencies elicited by angry facial expressions moderated this effect. Together, these findings may suggest that healthy female S-allele carriers do not only show more aggression in a laboratory aggression paradigm, but also that the magnitude of threat-avoidance tendencies might increase the amount of aggression displayed by female S-allele carriers and vice versa for L-allele homozygotes.

The genotype effect on aggression was specifically driven by the selected duration of white noise in the TAP. Interestingly, intensity and duration level selection on the TAP are considered to represent explicit and implicit aggression, respectively (Berkowitz, 1974; Carlson et al., 1989; Giancola and Zeichner, 1995). The clear effect of duration in our study suggests an effect of implicit aggression in these healthy females which is in line with previous findings showing that preadolescent females show more implicit aggression (Gonda et al., 2009). This contrasts with preadolescent males who tend to engage more in high levels of explicit aggression (Lagerspetz et al., 1988; Böjrkqvist et al., 1992). Our findings are also in line with previous investigations of implicit versus explicit aggression on the TAP after alcohol consumption, demonstrating that females increased implicit aggression only under the influence of alcohol, whereas males showed increases in both implicit and explicit aggression after alcohol consumption (Giancola and Zeichner, 1995). With the present study, we confirm and extend this previous work by showing that implicit aggression in females cannot only be reliably assessed by the TAP (Carlson et al., 1989; Giancola and Zeichner, 1995; Anderson and Bushman, 1997), but that it is also predominantly reflected in S-allele carriers.

The increased TAP aggression for S-allele carriers as compared to L-allele homozygotes is in line with the majority of literature suggesting an association with reactive aggression, although this is mainly shown in self-reported questionnaires and upon psychiatric evaluation (Hallikainen et al., 1999; Cadoret et al., 2003; Gerra et al., 2005; Sakai et al., 2006; Gonda et al., 2009; Retz and Rösler, 2009, but see Vassos et al., 2014). Interestingly, the short allele-variant of the 5-HTTLPR polymorphism has been associated with an increased risk to develop psychopathologies in combination with significant life events (Caspi et al., 2003; Kenna et al., 2012; Aleknaviciute et al., 2018; Saul et al., 2018) and this association seems to depend on sex differences (Du et al., 2000; Grabe et al., 2005; Sjöberg et al., 2006). Furthermore, a disrupted serotonin response has been associated with reactive aggression in particular (Coccaro and Kavoussi, 1997) (see for reviews, Duke et al., 2013; Coccaro et al., 2014). Our findings are therefore generally in line with the notion that serotonin dysfunction may be coupled to reactive aggression.

Most critically and in line with our hypothesis, the link between the S-allele carrying genotype and reactive aggression was moderated by threat-avoidance tendencies. This hypothesis was grounded in the notion that reactive aggression may be an expression of fear-driven defense, in contrast to instrumental aggression that has been associated with fearlessness (Blair et al., 2006; Cima and Raine, 2009; Lilienfeld et al., 2012). Our findings also fit notions from previous neuroimaging studies as S-allele carriers have repeatedly been shown to display increased amygdala activity in response to negative stimuli (Hariri et al., 2002; Heinz et al., 2005; Munafò et al., 2008; Blok et al., 2015). This suggests an increase in emotional reactivity to threat in S-allele carriers, which could influence fear-driven aggressive behavior. In the AAT, the tendency to avoid negative and approach positive stimuli might be triggered by the amygdala (Quirk et al., 2003; Volman et al., 2011, 2013). Therefore, amygdala activity during threat avoidance of angry faces might be increased in S-allele carriers, which could attribute to their increased reactive aggression. In line with the notion that reactive aggression stems from a lack of inhibitory control (Nelson and Trainor, 2007), earlier work showed that S-allele carriers display reduced connectivity between prefrontal control regions and the amygdala when controlling emotional action tendencies on the AAT (Volman et al., 2013). Therefore, poorly controlled amygdala-activity in S-allele carriers could amplify the tendency to avoid threat, which in turn can lead to reactive aggression. Our findings of increased threat-avoidance tendencies moderating the link between 5-HTTLPR genotype and reactive aggression (TAP), therefore supports existing theories that reactive aggression may be an expression of fearful behavior (Blair et al., 2006; Cima and Raine, 2009; Lilienfeld et al., 2012).

A few limitations should be considered when evaluating these findings. First, for reasons beyond the current research-question, we only included female participants, and it would be relevant to test these findings also in men. The S-allele 5-HTTLPR genotype in females is associated with more internalizing behavior, while in males this association is found with externalizing behavior (Gressier et al., 2016). In addition, it would be interesting to replicate these findings in a more heterogeneous participant sample, as the current homogeneity might have caused reduced statistical power to detect effects. Furthermore, future studies should take into account that the set order in which the tasks were completed could induce a carry-over effect from one to the other. Next, following the moderation analysis, the trend significant effects of the association between increased avoidance of angry faces on the AAT with increased reactive aggression on the TAP in S-allele carriers and decreased reactive aggression on the TAP in L-allele homozygotes do not provide definitive conclusions and should mainly encourage future work. Furthermore, our hypothesis for S-allele carriers was based on our previous fMRI study showing altered prefrontal-amygdala connectivity during the AAT in S-allele carriers (Volman et al., 2013). To adhere strictly to this hypothesis, we focused on the 5-HTTLPR polymorphism only. It would be interesting for future research to investigate polygenic risk scores for serotonin-related genes or to conduct cluster analysis in the serotonin spectrum. Therefore, this study should be evaluated as hypothesis-generating, and future work is needed to make the data conclusive. Finally, a strength of the current study is the use of objective measures for approach–avoidance tendencies as well as provoked reactive aggression. Nevertheless, it would be important for future investigations to replicate these findings, ideally combined with neuroimaging, to verify to what extend neural mechanisms typically underlying AAT-effects, link to neural mechanisms involved in the implicit aggression provoked by the TAP in S-allele carriers.

## Conclusion

Healthy female S-allele carriers seem to show more implicit reactive aggression during an aggression provocation, by selecting increased duration of white noise as a punishment for their alleged opponent on the TAP. This effect was moderated by avoidance tendency toward angry facial expressions on the AAT. These findings indicate that evaluative impulses in response to social cues play an important role mediating the genetic predisposition of the 5-HTTLPR polymorphism to increased expression of reactive aggression.

## Data Availability Statement

The raw data supporting the conclusions of this article will be made available by the authors, without undue reservation, on reasonable request.

## Ethics Statement

The studies involving human participants were reviewed and approved by local ethical review board (CMO region Arnhem-Nijmegen; NL42229.091.12). The patients/participants provided their written informed consent to participate in this study.

## Author Contributions

All authors listed have made a substantial, direct and intellectual contribution to the work, and approved it for publication.

## Conflict of Interest

BF received educational speaking fees from Medice. The remaining authors declare that the research was conducted in the absence of any commercial or financial relationships that could be construed as a potential conflict of interest.

## Footnotes

1 www.unipark.com
