# Supplementary material for: Threat-Avoidance Tendencies Moderate the Link Between Serotonin Transporter Genetic Variation and Reactive Aggression
Source: Front Behav Neurosci. 2020 Sep 28;14:562098. doi: 10.3389/fnbeh.2020.562098 (PMC7549659; doi:10.3389/fnbeh.2020.562098)
Supplement: Supplementary file 1 [file Table_1.docx]

**Supplementary table 1.** Mean (standard deviation; SD) scores in SRP (self-report psychopathy scale), TriPM (triarchic psychopathy measure), PPI (psychopathic personality inventory), LSAS (Liebowitz social anxiety scale), STAXI (state trait anger expression inventory) and STAI (state trait anxiety inventory) for participants in the S-allele carrier group and the L-allele homozygote group of the serotonin transporter-linked promoter region (5-HTTLPR) genotype.

|  | 5-HTTLPR genotype | |
| --- | --- | --- |
|  | S-allele carriers (*N*=55) | L-allele homozygote (*N*=30) |
| SRP Total | 48.96 (12.18) | 45.70 (10.14) |
| TriPM |  |  |
| Boldness | 27.89 (7.13) | 31.00 (6.81) |
| Meanness | 7.51 (6.31) | 7.60 (5.92) |
| Disinhibition | 9.87 (5.99) | 10.20 (7.34) |
| PPI Total | 332.24 (42.26) | 325.33 (36.30) |
| LSAS |  |  |
| Anxiety | 40.80 (8.04) | 40.73 (8.61) |
| Avoidance | 39.22 (8.91) | 37.40 (7.34) |
|  | S-allele carriers (*N*=62) | L-allele homozygote (*N*=30) |
| STAXI |  |  |
| State | 10.87 (2.81) | 10.30 (0.91) |
| Trait | 14.40 (2.99) | 14.33 (3.63) |
| STAI |  |  |
| State | 35.69 (9.23) | 34.12 (8.75) |
| Trait | 35.69 (8.47) | 33.60 (8.97) |
