# Supplementary material for: Threat-Avoidance Tendencies Moderate the Link Between Serotonin Transporter Genetic Variation and Reactive Aggression
Source: Front Behav Neurosci. 2020 Sep 28;14:562098. doi: 10.3389/fnbeh.2020.562098 (PMC7549659; doi:10.3389/fnbeh.2020.562098)
Supplement: Supplementary file 2 [file Table_2.docx]

**Supplementary table 2.** Correlations between the RPQ, IPAS and TAP subscales for reactive and instrumental aggression. Significant correlations indicated by * (p<0.05).

|  | RPQ Reactive aggression | RPQ Instrumental aggression | IPAS Impulsive aggression | IPAS Premeditated aggression | TAP before provocation | TAP after provocation | TAP provocation |
| --- | --- | --- | --- | --- | --- | --- | --- |
| RPQ Reactive aggression | - | r_s_  =.451 (p<.001)* | r_s_=.089 (p=.417) | r_s_=.281 (p=.045)* | r_s_=-.040 (p=.713) | r_s_=-.127 (p=.248) | r_s_=-.175 (p=.108) |
| RPQ Instrumental aggression |  | - | r_s_=.136 (p=.216) | r_s_=.218 (p=.045)* | r_s_=.085 (p=.439) | r_s_=-.074 (p=.498) | r_s_=-.036 (p=.744) |
| IPAS Impulsive aggression |  |  | - | r_s_=.110 (p=.315) | r_s_=-.037 (p=.733) | r_s_=.039 (p=.725) | r_s_=.018 (p=.870) |
| IPAS Premeditated aggression |  |  |  | - | r_s_=-.056 (p=.611) | r_s_=-.084 (p=.442) | r_s_=-.086 (p=.433) |
| TAP before provocation |  |  |  |  | - | r_s_=.736 (p<.001)* | r_s_=.735 (p<.001)* |
| TAP after provocation |  |  |  |  |  | - | r_s_=.811 (p<.001)* |
| TAP provocation |  |  |  |  |  |  | - |
